# Examining the types and payments of the disabilities of the insurants in the national farmers' health insurance program in Taiwan

**DOI:** 10.1186/1471-2458-10-646

**Published:** 2010-10-26

**Authors:** Jiun-Hao Wang, Hung-Hao Chang

**Affiliations:** 1 Department of Bio-industry Communication and Development, National Taiwan University, Taiwan; 2 Department of Agricultural Economics, National Taiwan University, Taiwan

## Abstract

**Background:**

In contrast to the considerable body of literature concerning the disabilities of the general population, little information exists pertaining to the disabilities of the farm population. Focusing on the disability issue to the insurants in the Farmers' Health Insurance (FHI) program in Taiwan, this paper examines the associations among socio-demographic characteristics, insured factors, and the introduction of the national health insurance program, as well as the types and payments of disabilities among the insurants.

**Methods:**

A unique dataset containing 1,594,439 insurants in 2008 was used in this research. A logistic regression model was estimated for the likelihood of received disability payments. By focusing on the recipients, a disability payment and a disability type equation were estimated using the ordinary least squares method and a multinomial logistic model, respectively, to investigate the effects of the exogenous factors on their received payments and the likelihood of having different types of disabilities.

**Results:**

Age and different job categories are significantly associated with the likelihood of receiving disability payments. Compared to those under age 45, the likelihood is higher among recipients aged 85 and above (the odds ratio is 8.04). Compared to hired workers, the odds ratios for self-employed and spouses of farm operators who were not members of farmers' associations are 0.97 and 0.85, respectively. In addition, older insurants are more likely to have eye problems; few differences in disability types are related to insured job categories.

**Conclusions:**

Results indicate that older farmers are more likely to receive disability payments, but the likelihood is not much different among insurants of various job categories. Among all of the selected types of disability, a highest likelihood is found for eye disability. In addition, the introduction of the national health insurance program decreases the likelihood of receiving disability payments. The experience in Taiwan can be valuable for other countries that are in an initial stage to implement a universal health insurance program.

## Background

The lack of health insurance coverage for many residents in rural areas is an important public health issue of rural development in many countries [1,2]. In the United States, for example, farmers are required to pay high premiums to purchase health insurance due to the higher risks in their professions. The medical bills they are unable to pay significantly affect family farms' overall financial stability. Healthcare expenses can possibly lead to increased credit card debt or loss of credit. For farmers and ranchers, healthcare expenses have the potential to affect not only their families' economic security but also the financial viability of their farm businesses [3-5]. To ensure the well-being of their farm population, many European countries, including Germany and France, introduced an occupational health insurance programs for farmers [6-8].

To promote the health of the farm population, the Taiwanese government has introduced a health insurance for farmers in particular. To remove the barriers to the access of medical care and improve equity in accessibility to medical resources for residents in rural areas, the Farmers' Health Insurance Program (FHI) was formally enacted in July 1989 [9]. The FHI, the first national social insurance system for agricultural workers in Taiwan, provides the farm population with a favorable premium-benefit ratio and high premium subsidy [10,11]. On average, the FHI program each year insures more than 1.7 million people who make up the agricultural population [12]. In the early years, the benefits of the FHI covered five risk categories: maternity, injury, sickness, disability, and death. After the inauguration of the National Health Insurance (NHI) program in 1995, all medical care benefits associated with injury and sickness in the FHI program were taken over by the NHI [10,13]. The benefits remaining in the FHI are: lump-sum cash payments related to the maternity subsidy, disability compensation and funeral allowance [9]. In particular, the expenditure in regard to disability payments has increased dramatically from the NTD 5.6 million (New Taiwanese Dollars; $US1 = 32 NTD Approx.) in 1989 to 3.7 billion in 2007. Since 2000, approximately 44%-65% of the cash payments were paid for disabled insurants in the FHI [12,14]. In order to receive disability payments, each insurant's degree of disability has to be diagnosed through the medical system. Therefore, the examination of the FHI insurants in Taiwan provides a good source of data to study the disabilities of the farm population.

A study on the disabilities of the farm population can also serve as reference for particular policy interests [15,16]. Although a growing body of literature has examined occupational risk within the general population [17-19], little attention has been paid to farmers or rural population [20,21]. This study aimed to fill this knowledge gap by using population-based insurants data of the FHI program in Taiwan. The primary objective of this study was to determine the disability types and payments arising from the FHI program in Taiwan. We examined how the social-demographic characteristics are associated with different types of disabilities and payments, and how the nature of the disabilities affects insurance payments. Special attention is paid to distinguish the extent to which age and different job identification of the insurants are associated with the probability of receiving disability payments. In contrast to previous studies [3,4,22,23], our study is unique in several ways. First, although the importance of farmers' health insurance and disability payments in rural development had already been recognized, little empirical evidence had been provided based on large-scale data. Differing from earlier studies that relied on the collection of data for a limited sample size or on restricted areas [24-27], our study utilizes a national insurants sample of the FHI program. Moreover, whereas the former studies on occupation risk usually focused solely on the association between different job-related practices and occupational risk in general [28-30], this study aimed to distinguish the effects of insured job categories and age on the likelihood and levels of disability payments among the insurants.

## Methods

### Data

We used a national insurants data file containing information on each insured person enrolled in the FHI program at the end of July 2008. This dataset was collected and provided by the Bureau of Labor Insurance (BLI) of the Council of Labor Affairs in Taiwan. The insurance data file was made up of the claims submitted monthly by each local Farmers Association (FA, the basic insured unit). We have permission to access the individual data under the academic project (Grant No. 98AM-5.7-FS-04) sponsored by the Council of Agriculture and the Bureau of Labor Insurance in Taiwan under the confidentiality agreement. The dataset contained detailed information on each insured person in the FHI program, such as job identification, and the year of enrollment. However, except for age and gender, not much information concerning the socio-demographic characteristics was documented. In total, 1,594,439 insurants in the FHI program were included in this study.

### Measures

Although there are three types of lump-sum cash benefits provided by the FHI program, we focused our analysis on disability payments. This is due to the death payments not being documented because the claims data set contains only those currently registered in the FHI program. While the spouse of the farm operator was also eligible for maternity payments, these only accounted for a small proportion (less than 4%) of the entire budget [12].

#### Socio-demographic characteristics

The socio-demographic characteristics of the insured persons included age and gender. Age was categorized into several dummy variables, including those less than 45 years old, as well as those in the 45-54, 55-64, 65-74, 75-84 age ranges, and those over 85. Furthermore, all insurants were divided into four geographic regions: the north, middle, south regions, and east area of Taiwan.

#### Insured job categories

Several job categories of the insurants were identified, including: self-employed farmers, tenants, spouses of the farm operators, as well as hired farm workers. In addition, all insurants could either compulsorily or voluntarily participate in the FHI program. The former category was limited to those farmers who had enrolled as members of the local farmers association (FA). Non-FA members could also participate in the FHI program if they qualified for the farming practical standards, such as being over the age of 15, having a minimal farm size of more than 0.1 hectares, and farming more than 90 days within one year [31]. Recognition of the different types of job categories is important since different job categories reflect different intensities of farm practice. For instance, spouses normally are farm helpers and they engage less frequently in farm practice. Therefore, they may encounter fewer occupational risks; in contrast, tenants and hired employees work on the farm more intensively. Therefore, they may have a higher likelihood of becoming disabled.

#### Types and payments of disability

Both the types and payments of disability are considered. With respect to the type of disability, in total, 10 types and 160 disability items are recognized by the FHI-Act [9]. The disability types, categorized into groups based on the affected part of the body, include mental and neuropathic, eyes, ears, nose, mouth, viscera in chest and abdomen, trunk, head- face- and neck, upper limbs, and lower limbs disability. Table 1 summarizes the detailed definition, content, and distribution of different disability types in the FHI. As exhibited, among all of the receipts of disability payments, the highest proportion of disability payments is for eye disabilities (45.5%); the lowest proportion is for nose problems (0.1%).

**Table 1:** Definition and distribution of different disability types in the FHI

Disability type	Disability Examination	%
*1. Mental and neurological disability*	The mental disability must be assessed in light of the Mini-Mental Status Examination (MMSE) or Wechsler Adult Intelligence Scale (WAIS) and issued by a psychiatrist. Neurological disability includes "dysfunction of balance and audio", epilepsy, headache, or "vertigo and balance" and so forth. Such disability must be issued by a specialist of the neurology, neurosurgery or rehabilitation departments.	17.5
*2. Eyes*	Eye disability includes eyesight, visual field, eyeball's regulating or moving, eyelid's defect, and movement disability.	45.5
*3. Ear*	Ear disability includes hearing disability of the inner and middle ear, auricle's defect disability.	1.7
*4. Nose*	Defect and functional disability of the nose.	0.1
*5. Mouth*	Mouth disability includes mastication, deglutition and speaking disability, and teeth disability.	1.4
*6. Viscera in chest and abdomen*	Viscera disability includes full or partial functionality damage to the thoracic, abdominal, urogenital viscera, and reproductive organs. The viscera disability cannot be judged until six months after medical treatment or the last surgery.	21.5
*7. Trunk*	Trunk disability includes rachis deformity or moving disability, and other trunk-bone deformity, such as breastbone, rib or blade bone.	9.5
*8. Head- face- and neck*	Head- face- and neck disability indicates ugly shape of the head, face and neck. For example, those who are damaged and left with obvious disfigurement on head or face.	0.6
*9. Upper limbs*	Upper limb disability includes upper limbs' or fingers' defective or functional disability, and deformity of homeruns or cubism.	0.5
*10. Lower limbs*	Lower limb disability includes lower limbs' or toes' defective or functional disability, shortening disability, and deformity of femur or tibia.	1.8

In total, 127,973 receipts of the disability payment from the FHI were included.

It is of note that the disability type is defined based on different body parts, and the type or category does not necessarily reflect the severity of the disability. The same disability type might have different degrees of disability. For example, an eye disability may include eyesight, visual field, and eyeball movement disabilities. The corresponding payments for these eye-related disabilities vary, and insurants who are totally blind receive higher payments. As a result, in addition to the disability types, we use the actual disability payment received by the insurants in the FHI program.

Disability compensation is a lump-sum payment ranging from NTD 10,200 to NTD 408,000, according to the level of disability. For example, the highest payments are for those with severe mental disabilities who need frequent medical protection and/or attention by a specially assigned individual. The lowest payments of disability are for those with the loss of the toes.

#### Identification of the National Health Insurance Program

The introduction of the National Health Insurance program in 1995 plays an important role in regard to disability payments. The criteria for receiving a diagnosis certificate of disability became stricter after the introduction of the NHI in 1995 because insured persons eligible for disability benefits had to receive a diagnosis certificate of permanent disablement from the NHI-contracted institutions. To capture the effects of the NHI program on the disability payments of insurants in the FHI, we employed a dummy variable indicating whether the farmers participated in the FHI before the NHI program (= 1 if they participated in the FHI before 1995).

### Statistical analysis

Our statistical analysis involved several steps. Firstly, we examined all the FHI insurants using the Pearson chi-square test to compare the distribution of social-demographic characteristics between those who had, and those who had never, received disability payments. In what follows, a logistic regression model was estimated to identify the odds ratios of insurants receiving the disability payments. We also used a multinomial logistic model to estimate the relationship between social-demographic and insured factors and the likelihood of having different types of disability. Additionally, we investigated how the socio-demographic and insured characteristics may have determined the level of disability payments by using the ordinary least squares regression model. All analyses were conducted using the statistical software Stata release 10 [32].

## Results

### Descriptive statistics of the FHI insurants

Table 2 presents the distribution of the socio-demographic characteristics for insurants who ever received disability payments and those who did not. Among the 1,594,439 insurants enrolled in the FHI program in 2008, 8% had received disability payments. The disability payments averaged NTD 103,730 (approximately equal to USD 3,231) per recipient. In general, those with disability payments were more likely to be older, female, self-employed, or those living in middle and southern areas. The results of the chi-square test showed that all the differences in selected socio-demographic characteristics and insured factors were statistically significant.

**Table 2:** Sample statistics by the recipients of disability payments

	With payments	No payments	Statistical Test*
		
Sample size	127,973(8%)	1,466,466(92%)	
Variables			p-value
*Average received disability payments (NTD)*^*$*^	103,730		
*If male farmer*	43%	52%	<0.001
*If participated before National Health Insurance*	79%	59%	<0.001
*Age groups*^*#*^			<0.001
<45	2%	12%	
45-54	7%	16%	
55-64	11%	18%	
65-74	20%	23%	
75-84	41%	22%	
> = 85	19%	9%	
*Insured job categories*			<0.001
Self-employed farmer, non-FA member	35%	42%	
Tenant, non-FA member	1%	1%	
Spouse of the farm operator	9%	7%	
Self-employed farmer, FA member	45%	42%	
Tenant, FA member	2%	2%	
Hired worker	8%	6%	
*Geographic region*			<0.001
North	9%	15%	
Middle	39%	42%	
South	47%	38%	
East	5%	5%	

* The Pearson chi-square test was used for the differences in sample means between these two groups.

1,594,439 insurants covered by the Farmers' Health Insurance on July 31, 2008 were included.

$ The exchange rate as of July 14, 2010 was 1 USD = NT$32.1 approximately.

# The average age of the insurants is 61.

### The likelihood of receiving disability payments

Table 3 shows the estimated odds ratios of the logistic regression model. Compared with female insurants, male insurants are less likely to receive payments. The odds ratio is 0.78. Insurants who participated in the FHI before the introduction of the NHI in 1995 were more likely to receive disability payments (odds ratio 1.64) compared to their counterparts. In addition, the positive correlation between age and the likelihood of receiving disability payments is evident. The effects are more pronounced for older insurants. For instance, compared with the enrollees less than 45 years old, the odds ratios of those who were 45-54 years old and above 85 were 2.41 and 8.04, respectively.

**Table 3:** Estimation of the probability of receiving disability payments

Variables	Odds Ratios	95% CI
*Male*	0.78	[0.77, 0.79]
*If participation before NHI program*	1.64	[1.62, 1.67]
*Age group*		
<45 (reference group)	1.00	
45-54	2.41	[2.33, 2.49]
55-64	2.72	[2.63, 2.81]
65-74	5.24	[5.08, 5.41]
75-84	8.45	[8.18, 8.72]
> = 85	8.04	[7.75, 8.34]
*Insured job categories*		
Self-employed farmer, non-FA member	0.97	[0.94, 0.99]
Tenant, non-FA member	1.07	[1.02, 1.13]
Spouse of the farm operator	0.85	[0.83, 0.88]
Self-employed farmer, FA member	0.93	[0.91, 0.95]
Tenant, FA member	1.06	[1.01, 1.11]
Hired worker (reference group)	1.00	
*Geographic region*		
North (reference group)	1.00	
Middle	1.72	[1.68, 1.76]
South	2.23	[2.18, 2.28]
East	1.78	[1.72, 1.83]

The odds ratios are measured by: with *vs. *without disability payments groups.

The chi-square test for the null hypothesis H_o_: all coefficients are zero is x^2^(15) = 66.8.

Insured job categories are also important factors influencing whether insurants receive disability payments. However, the odds ratios among different job categories did not differ to a significant degree. For instance, compared with the hired workers (the reference group), the odds ratios of self-employed farmers of non-FA members and their spouses were 0.97 and 0.85, respectively. In contrast, the likelihood of the tenants of FA and non-FA members receiving disability payments is higher than for the hired workers. The odds ratios of these two groups of insured persons are 1.06 and 1.07, respectively. Finally, regional heterogeneity is also significantly associated with the likelihood of receiving disability payments. Compared with the insured persons living in the northern area (the reference group), the odds ratios for those in the middle, southern and eastern areas were 1.72, 2.23 and 1.78, respectively.

### The likelihood of different types of disability

Table 4 presents the estimated odds ratios of the multinomial logistic model. Among the ten types of disability, we chose eye disability (type 2 in Table 1) as the reference group owing to its representing the highest proportion of payments (45.5% of all recipients of the payments). In general, compared to their counterparts, females, the elderly, those who had joined the FHI before 1995, or had lived in non-northern areas were more likely to obtain a medical certificate for eye disability than for any other type of disability. In general, male insurants were more likely than their counterparts to have a non-eye-related disability. For instance, the odds ratio of male insurants having a mouth-related disability rather than an eye-related disability (type 5 vs. type 2) increased by a factor of 11.044, given all other variables remain constant.

**Table 4:** Estimated Odds Ratios of the Multinomial logistic model of disability types

Type*	1 vs. 2	3 vs. 2	4 vs. 2	5 vs. 2	6 vs. 2	7 vs. 2	8 vs. 2	9 vs. 2	10 vs. 2
*Male*	3.396**	**2.838**	1.787	**11.044**	1.012	**0.861**	**6.233**	**2.288**	**1.728**
*If before NHI*	**1.078**	**1.273**	**0.406**	**1.326**	**1.084**	**1.108**	1.179	1.043	**1.441**
*Age group*									
<45 (ref. group)	1.000	1.000	1.000	1.000	1.000	1.000	1.000	1.000	1.000
45-54	**0.426**	1.237	0.532	1.042	1.084	**0.585**	**0.367**	0.592	0.617
55-64	**0.095**	**0.473**	**0.063**	**0.175**	**0.085**	**0.354**	**0.045**	**0.140**	**0.120**
65-74	**0.024**	**0.165**	**0.007**	**0.021**	**0.007**	**0.121**	**0.005**	**0.031**	**0.037**
75-84	**0.012**	**0.109**	**0.001**	**0.007**	**0.003**	**0.059**	**0.001**	**0.011**	**0.028**
> = 85	**0.008**	**0.133**	**0.002**	**0.005**	**0.002**	**0.043**	**0.001**	**0.007**	**0.024**
*Insured job categories*									
Self-employed farmer, non-FA	1.028	0.853	2.169	0.904	0.922	1.091	0.778	1.584	1.010
Tenant, non-FA	**1.322**	0.652	7.802	0.799	0.957	1.152	1.056	2.297	**1.771**
Spouse of the farm operator	**1.138**	0.978	**9.223**	1.191	**1.274**	**1.485**	0.896	**2.052**	1.085
Self-employed. farmer, FA	0.927	0.838	3.284	0.858	**1.214**	1.045	**0.671**	1.321	0.898
Tenant, FA	1.153	1.228	8.719	0.894	**1.381**	**1.382**	**0.388**	**2.285**	**1.618**
Hired worker (ref. group)	1.000	1.000	1.000	1.000	1.000	1.000	1.000	1.000	1.000
*Geographic region*									
North(ref. group)	1.000	1.000	1.000	1.000	1.000	1.000	1.000	1.000	1.000
Middle	**0.502**	**0.343**	0.955	**0.689**	**0.457**	**0.799**	**0.440**	**0.331**	0.921
South	**0.424**	**0.247**	0.470	**0.421**	**0.358**	**0.692**	**0.394**	**0.273**	**0.535**
East	**0.508**	**0.716**	0.558	0.817	**0.419**	**0.721**	**0.412**	0.638	1.206

* Type 1 = Mental and neurological; 3 = Ear; 4 = Nose; 5 = Mouth; 6 = Viscera disability in chest and abdomen; 7 = Trunk; 8 = Head- face- and neck; 9 = Upper limbs; 10 = Lower limbs. Eye disability (Type 2) the reference group. Detailed definitions of disability types can be found in Table 1.

** The bold number indicates statistical significance at least 5% level.

The odds ratios are measured by: Diagnosis of other disability types vs. diagnosis of eye disability.

The introduction of the NHI has also played an important role in the receipt of payment for different types of disability. In general, those who enrolled in the FHI before 1995 were less likely to be eye disabled compared to different types of disability. For instance, compared with their counterparts, the odds ratios of those who participated before the NHI for mental and neurological (type 1), ear (type 3), trunk (type 7), and lower limbs (type 10) disabilities are 1.078, 1.273, 1.108, and 1.441, respectively (compared to eye disability, type 2). Finally, regional differences are significant among disability types, and most obviously between the northern and other geographic regions. If one considers trunk versus eye disability (type 7 vs. type 2) for example, those insurants who live in the middle, south and east had greater likelihood of acquiring a trunk-related disability than did northern insurants, the odds ratios are: 0.799, 0.692, and 0.721, respectively.

Among all of the determinants, we found age to be the most significant factor contributing to the type of disability. Compared to insurants younger than age 45 (the reference group), those who were older are more likely to have eye disability and less likely to have other types of disability. For example, compared with those less than 45, the odds ratios of having a trunk disability versus an eye disability for those 45-54 years old and above 85 were 0.585 and 0.043, respectively.

With respect to the insured job categories, the effects do not differ significantly across different types of disability. In general, farm spouses were more likely to have mental and neurological (type 1), nose (type 4), viscera (type 6), trunk (type 7), and upper limb (type 9) disabilities than eye disabilities (type 2) in comparison to hired workers. In addition, a higher likelihood of having viscera (type 6), trunk (type 7), upper limb (type 9), and lower limb (type 10) disabilities, compared to an eye problem, is evident for tenants (FA member); the odds ratios are: 1.381, 1.382, 2.285, and 1.618, respectively.

### Determinants of the level of disability payments

Table 5 presents the results of the ordinary linear square regression method, with the focus on the recipients of the level of disability payments. In Table 5, the dependent variable is the amount of disability payments that each insurant received. It is noteworthy that in the preliminary analysis, we estimated a log-normal payment equation as well. However, due to the ease of interpretation and the qualitatively consistent results between these two models, we report the results of only the linear payment equation here. As exhibited in Table 5, compared with female recipients, males received NTD 434 less on average. For those who participated in the FHI program before 1995, they received NTD 2,947 more compared to the other participants. The relationship between age and disability payments was also found to be significant, and was more pronounced among older recipients. For instance, recipients aged 85 and above received NTD 15,130 more than those who were less than 45 years old.

**Table 5:** Estimation of the receipts of the disability payments in the FHI program

Variables	Coefficients	Std. Err.	P-value
*Male*	-434	57	<0.001
*If participated before NHI*	2,947	58	<0.001
*Age group*			
<45 (reference group)			
45-54	1,521	94	<0.001
55-64	3,064	95	<0.001
65-74	7,844	88	<0.001
75-84	13,716	97	<0.001
> = 85	15,130	147	<0.001
*Insured job categories*			
Self-employed farmer, non-FA member	-526	116	<0.001
Tenant, non-FA member	102	247	0.67
Spouse of the farm operator	-1,446	151	<0.001
Self-employed farmer, FA member	-910	116	<0.001
Tenant, FA member	188	214	0.38
Hired worker (reference group)			
*Geographic region*			
North (reference group)			
Middle	2,918	81	<0.001
South	4,761	82	<0.001
East	3,251	135	<0.001
*Constant*	-1,798	150	<0.001

The exchange rate as of July 14, 2010 was 1 USD = NT$32.1 approximately.

The evidence also pointed to the significant relationship between disability payments and those who were self-employed farmers or the spouses of farm operators. Compared with hired workers, the self-employed farmers (FA members and non-FA members) and the spouses of farm operators each received NTD 526, NTD 1,446, and NTD 910 less on average, respectively. Finally, insurants in different geographic regions also received different amounts. Compared with those living in the northern area of Taiwan, those residing in the middle, southern, eastern areas and the offshore islands of Taiwan on average received NTD 2,918, NTD 4,761, NTD, and NTD 3,251 more in terms of disability payments, respectively.

## Discussion and conclusions

Studying disability is of particular policy interest since it is one of the important determinants of the quality of life for the rural elderly [33]. By using a unique dataset for all of the insurants registered in the FHI program in July 2008 in Taiwan, we investigated the extent to which socio-demographic factors and job categories may be associated with the likelihood of receiving disability benefits. Furthermore, we examined how these factors are associated with the types and payments of disability. To the best of our knowledge, there are limited studies to address this issue by using a population-based insurants dataset.

Several interesting findings may be noted. First, age and job categories were found to be significantly associated with the disability benefits in the FHI program. Results indicate that older farmers are more likely to receive payments. However, the likelihood of receiving disability payments does not differ significantly among insurants of various insured job categories. Although our analysis is only for the insurants in the FHI program, the results may demonstrate that the natural causes related to age structure are the main driving factors of the disability incidence among the FHI insurants. If eye disability reflects natural causes of disability, we find that older insurants are more likely not only to receive higher payments, but also payments related to eye problems. Several implications of public health policy can be inferred from this finding. For example, older insurants with eye disability appear to be more dependent outside of the home and in need of help with mobility. To increase their capacity of independence outside of the home, it may be necessary to provide more direct assistance in the local areas of mobility. Furthermore, from the preventive point of view, eye disability can be possibly prevented by regular eye examination to increase the frequency of screening visual acuity. Improving the training programs of the practice nurses or optometrists will also help.

Geographic residence also matters. Insured persons in the southern area received more payments compared to those living in other areas. This was consistent with the fact that the major agricultural production area is located in the southern part of Taiwan. Interestingly, a crowding-out effect of the NHI program on the FHI is also evident. After the implementation of the National Health Insurance program in 1995, the various health authorities of different occupational insurance systems were integrated into a single mechanism [10,13]. The medical diagnoses and treatments for disability had to be approved by the National Health Insurance authorized clinical system. Therefore, the unified screening system of the National Health Insurance reduced the likelihood of receiving disability payments in the FHI.

Consequently, the strength of this study lies in using data from a large population-based survey to examine the disability payments of the National Farmers' Health Insurance program in Taiwan. This topic is relevant to current global discussions on health insurance models in rural communities; however, there are some limitations inherent in the present study. For instance, the information on the socio-demographic characteristics is limited; this is one of the common problems of using large-scale insurant data. Our findings could be more significant if other social-economic characteristics, such as health status, education, income and farming activities of each insurant, were available. In addition, as pointed out by one of the anonymous reviewers, our study subjects include all of the insurants in the FHI program, and this is not necessarily reflect that they are all working in the farm-related activities. Therefore, further detailed information related to farm activities can be helpful to show the robustness of our findings. Moreover, the highest probability of disability type is found for eye disability. Further research can investigate the extent to which the eye disability is associated with aged-related, environmental, infectious, and occupational factors.

## Abbreviations

BLI: Bureau of Labor Insurance; FA: Farmer's Association; FHI: Farmers' Health Insurance Program; NHI: National Health Insurance

## Competing interests

The authors declare that they have no competing interests.

## Authors' contributions

JHW drafted the manuscript, coordinated the data collection, and participated in the design of the study. HHC contributed to the conceptual framework, designed the study, revised the manuscript, and carried out the data analysis. All of the authors read and approved the final manuscript.

## Pre-publication history

The pre-publication history for this paper can be accessed here:

http://www.biomedcentral.com/1471-2458/10/646/prepub
